# Nucleotide signaling in neutrophils: a key player in cancer dynamics

**DOI:** 10.1007/s11302-026-10166-2

**Published:** 2026-05-25

**Authors:** Jean Lucas Gutknecht da Silva, Jean Sévigny, Daniela Bitencourt Rosa Leal

**Affiliations:** 1 Laboratory of Experimental and Clinical Immunobiology, Department of Microbiology and Parasitology, Federal University of Santa Maria, Santa Maria, Brazil; 2 Axe maladies infectieuses et immunitaires, Centre de recherche du CHU de Québec, Université Laval, Québec, QC G1V 4G2 Canada; 3 Département de microbiologie-infectiologie et d’immunologie, Centres PROTEO et ARThrite, Faculté de Médecine, Université Laval, Québec, QC G1V 0A6 Canada

**Keywords:** Neutrophils, Purinergic signaling, Cancer, Therapeutic target

## Abstract

Neutrophils are key innate immune cells whose effector mechanisms are increasingly recognized as therapeutic targets in chronic inflammation and cancer. While highly heterogeneous and functionally versatile, they can also exacerbate inflammation and tissue damage, making precise modulation critical for homeostasis. Nucleotide signaling—including extracellular nucleotide-metabolizing enzymes and receptors for ATP, UTP, and adenosine—plays a central role in regulating neutrophil responses. Extracellular nucleotides influence adhesion, chemotaxis, and degranulation, with important implications in cancer. Neutrophil heterogeneity affects tumor progression through both pro- and antitumor mechanisms. This review summarizes these findings and highlights therapeutic strategies targeting nucleotide signaling to modulate neutrophil activity in cancer.

## Introduction

Nucleotide signaling has been extensively studied in human cells and is recognized as a key therapeutic target in several diseases, including cancer [1]. Neutrophils play essential roles in inflammation, including adhesion, phagocytosis, degranulation, and neutrophil extracellular trap (NET) formation, all of which contribute to the regulation of cancer progression. Notably, neutrophils are abundant within the tumor microenvironment (TME) and can adopt a pro-tumor phenotype. These cells express nucleotide-responsive receptors, which may offer novel opportunities to modulate their activation under different pathological conditions. In this review, we discuss the potential roles of nucleotide signaling in neutrophil function, focusing on its impact on tumorigenesis and cancer progression.

## An overview of neutrophils

Neutrophils constitute a central component of innate immunity and are the first leukocytes recruited to sites of inflammation or infection [2]. A wide range of neutrophil functions can be evaluated using in vitro and ex vivo biochemical approaches, including calcium flux, F-actin polymerization, adhesion, aggregation, degranulation, phagocytosis, and reactive oxygen species (ROS) production. In vivo, neutrophil activity can be assessed through processes such as NET formation, recruitment, and migration. Notably, many of these functional responses are accompanied not only by biochemical changes but also by pronounced alterations in the biophysical properties of neutrophils, including cell shape, size, deformability, and other mechanical characteristics [3].

Following intravenous lipopolysaccharide (LPS) challenge, pulmonary neutrophils exhibit increased surface expression of CD11b and rapidly adopt a crawling phenotype along the vascular endothelium. This behavior depends on Toll-like receptor 4 (TLR4), myeloid differentiation primary response 88 (MyD88), and Abl kinase signaling, and appears independent of passive physical trapping. Neutrophil recruitment is initiated by interactions between selectins and their glycosylated ligands, specifically P- and E-selectin binding to P-selectin glycoprotein ligand 1 (PSGL-1), which mediates neutrophil tethering and rolling along the endothelium. In contrast, L-selectin does not directly mediate primary tethering but promotes secondary tethering of free-flowing neutrophils to those already rolling. Subsequent deceleration and firm arrest are achieved through interactions between lymphocyte function–associated antigen 1 (LFA-1) and intercellular adhesion molecules (ICAM-1 and ICAM-2). During this process, integrins undergo conformational changes from a low- to a high-affinity state, enabling stable adhesion to endothelial immunoglobulin superfamily adhesion molecules. Firm adhesion via LFA-1–ICAM-1 interactions is essential for the next stage of recruitment, during which neutrophils extend pseudopodia across the endothelial surface and initiate active motility and transmigration [4].

Upon arrival at sites of tissue damage or infection, neutrophils execute a range of antimicrobial effector functions, including phagocytosis, degranulation, and NET release [2]. Two mechanistically distinct forms of NET formation have been described: lytic NETosis (classical or suicidal NETosis), a specialized form of programmed cell death distinct from apoptosis and necrosis. This pathway is frequently dependent on NADPH oxidase–derived ROS, which activate PAD4 (peptidylarginine deiminase 4), promoting histone citrullination and chromatin decondensation. Nuclear translocation of neutrophil elastase (NE) and myeloperoxidase (MPO) further facilitates chromatin unraveling, culminating in plasma membrane rupture and extrusion of DNA ornamented with granular proteins. Beyond antimicrobial activity, this form of NET release contributes to tissue injury, persistent inflammation, and genomic instability, and has been associated with tumor growth and metastatic dissemination in multiple models [5]; and vital NETosis in which neutrophils remain viable and retain key cellular functions following NET release [6]. It can be triggered by microbial or inflammatory stimuli through receptors such as TLR2, TLR4, or complement receptors, leading to PAD4-mediated chromatin decondensation, often with reduced dependence on oxidative burst. Instead of membrane rupture, chromatin is expelled via vesicular trafficking, preserving membrane integrity and allowing neutrophils to retain functions such as phagocytosis and cytokine production. In the TME, both lytic and vital NET formation have been implicated in cancer progression, including enhancement of tumor cell proliferation, awakening of dormant cells, extracellular matrix remodeling, and facilitation of metastatic seeding [5].

Among neutrophil subsets, low-density neutrophils (LDNs) have emerged as a population of particular interest due to their pronounced immunosuppressive properties in cancer. LDNs consistently display elevated expression of activation markers, including CD11b, CD16, CD35, and CD66b. In oncological settings, these cells exhibit enhanced NET formation, increased arginase-1 (ARG1) production, and potent immunosuppressive activity, notably through inhibition of T-cell proliferation [2].

TME is a complex and dynamic system comprising malignant cells along with diverse stromal and immune components, including lymphocytes, dendritic cells, macrophages, endothelial cells, fibroblasts, and extracellular matrix constituents such as hyaluronic acid, fibronectin, laminin, and collagen.

Within this context, neutrophils are also present and display diverse functional responses depending on their phenotypic alterations, reflecting a highly heterogeneous and plastic cell population. Terms such as polymorphonuclear neutrophils (PMNs), high- or low-density neutrophils (HDNs and LDNs), tumor-associated neutrophils (TANs), and myeloid-derived suppressor cells (MDSCs) are commonly used to describe subpopulations of what is fundamentally the same cell lineage [7].

Depending on local stimuli, soluble mediators in the microenvironment, the functional demands imposed on them, and morphological features that are not yet fully standardized, these neutrophils can acquire either pro-tumorigenic or anti-tumorigenic properties [8].

TANs are recruited to and shaped by cytokines and chemokines within the TME. These cells exhibit marked functional heterogeneity and can be distinguished according to their surface marker profile and their capacity either to restrain or to promote tumor growth [9]. Similar to the M1/M2 macrophage paradigm, TANs are classified into two major phenotypes: N1 and N2 [10].

Although the N1/N2 framework has been proposed to distinguish tumor-inhibiting from tumor-promoting neutrophils, supporting evidence in human cancer remains limited, and their defining features including surface markers, cytokine profiles, and transcriptional regulators are not fully established. Current data suggest that these phenotypes do not represent rigid or discrete states, but rather a dynamic and context-dependent continuum. Therefore, in this review, the N1/N2 terminology is used as a conceptual tool to facilitate discussion of the pro-tumoral and antitumoral functions that neutrophils may assume across different microenvironmental conditions.

The N1 subset displays antitumor properties and is characterized by elevated production of pro-inflammatory and immunostimulatory mediators, including TNF-α, CCL3, and ICAM-1, along with reduced expression of the immunosuppressive ARG1. In contrast, N2 TANs exhibit a pro-tumor phenotype, with increased expression of chemokines such as CCL2, CCL3, CCL4, CCL8, CCL12, CCL17, and CXCL1, CXCL2, CXCL8, and CXCL16 [10, 11], as well as higher ARG1 levels. Moreover, N2 cells support tumor-associated angiogenesis through the recruitment of matrix metalloproteinases (MMPs) [12].

Circulating (peripheral) neutrophils also demonstrate functional polarization and are commonly categorized based on density into high-density neutrophils (HDNs) and low-density neutrophils (LDNs) [13]. The LDN fraction consists of a heterogeneous mixture of mature and immature cells and is associated with altered functional properties, including the generation of ROS, nitric oxide, and ARG1, as well as the induction of regulatory T cells (Tregs) [7]. Accumulating evidence indicates that elevated LDN levels correlate with enhanced immunosuppressive mechanisms and are positively associated with tumor progression in both murine models and human malignancies [7]. Experimental data further suggest that HDNs and LDNs exert opposing roles during cancer development and progression.

Neutrophil polarization is a pivotal mechanism underlying the emergence of distinct functional subsets that directly impact tumor growth and progression. Owing to their pronounced plasticity, neutrophils can dynamically shift their phenotype in response to cytokines and additional signals within the tissue microenvironment. IFN-β and transforming growth factor-β (TGF-β) signaling pathways constitute major regulatory axes governing the differentiation toward N1 and N2 phenotypes, respectively, and therefore represent strategic targets for modulating antitumor immune responses. Evidence indicates that the absence of IFN-β promotes the expansion of N2-like neutrophils, which are associated with reduced tumor cell cytotoxicity, diminished NET formation, and lower expression of ICAM-1 and TNF-α, highlighting the role of IFN-β in driving N1 polarization [14]. Conversely, IL-35 and TGF-β favor the acquisition of the N2 phenotype, enhancing tumor infiltration and disease progression. Pharmacological inhibition of TGF-β signaling, either through receptor blockade or neutralizing antibodies, has been shown to suppress tumor cell proliferation by modulating Smad and PI3K/AKT pathways in colorectal cancer models [10, 15].

MDSCs comprise a heterogeneous population of pathologically activated immature myeloid cells characterized by potent immunosuppressive activity. These cells expand under inflammatory conditions, including experimental cancer, and are broadly categorized into granulocytic (PMN-MDSCs) and monocytic (M-MDSCs) subsets [16]. Notably, increased levels of PMN-MDSCs in cancer patients have been consistently associated with poor clinical outcomes [17, 18].

Distinguishing PMN-MDSCs from circulating neutrophils remains challenging because they share overlapping surface markers in both murine models and humans. Even markers proposed to provide greater specificity, such as LOX-1 and CD10, have not proven sufficiently reliable to clearly discriminate between these populations [19, 20]. Although PMN-MDSCs were initially defined as immature myeloid cells with immunosuppressive properties, activated mature neutrophils can also suppress T-cell responses. This functional convergence, combined with the marked plasticity of neutrophils within the TME, further complicates precise classification.

In response to signals derived from the TME, neutrophils may adopt immunosuppressive and pro-tumorigenic phenotypes, often referred to as “immunosuppressive neutrophils.” For clarity, throughout this context we will preferentially refer to neutrophils associated with tumors as TANs, subclassified as N1 when exhibiting antitumor activity and N2 when displaying pro-tumor functions.

TANs are frequently polarized toward pro-tumorigenic phenotypes, promoting immunosuppression, angiogenesis, metastatic dissemination, and resistance to anticancer therapies. As critical components of the pre-metastatic niche, neutrophils contribute to the establishment of a pro-angiogenic tumor milieu by secreting soluble mediators, including MMPs, vascular endothelial growth factor (VEGF), TGF-β, and interleukin-17 (IL-17). These factors sustain chronic inflammation, exacerbate immunosuppressive signaling, stimulate angiogenesis, and drive extracellular matrix remodeling, collectively facilitating tumor progression [21]. Additionally, TANs have been implicated in modulating therapeutic responses of non-small cell lung cancer (NSCLC) to immune checkpoint inhibitors targeting programmed cell death protein 1 (PD-1) and its ligand PD-L1 via phenotypic reprogramming [22].

Despite these advances, the mechanisms underlying TAN reprogramming—from predominantly immunosuppressive states to profiles that support antitumor immunity during immunotherapy—remain incompletely understood. Emerging evidence suggests that coordinated action of type I and type II interferons is required to effectively reprogram TANs toward an antitumor functional state [23].

## An overview of nucleotide signaling in neutrophils

Beyond their classical immunological functions, neutrophil behavior is tightly regulated by extracellular signals, among which purinergic signaling has emerged as a key modulatory system. Nucleotide signaling involves extracellular messengers such as ATP, ADP, UDP, and the nucleoside adenosine (ADO), which activate P2 and P1 receptors, respectively. These molecules mediate autocrine and paracrine signaling by binding to their corresponding receptors. The process is regulated by ectonucleotidases, enzymes that hydrolyze nucleotides. Specifically, ectonucleoside triphosphate diphosphohydrolase-1 (NTPDase1 or CD39) and ecto-5′-nucleotidase (CD73) convert ATP/ADP to AMP, and AMP to ADO, respectively, thereby controlling the availability of substrates and products for downstream signaling [24]. Cell surface-located NTPDases are of major importance for controlling the availability of extracellular nucleotide agonists at P2 receptors. They also contribute to recycling of nucleosides derived from extracellular nucleoside phosphates and metabolic salvage pathways [25].

Neutrophils express both ectonucleotidases and P1/P2 receptors, which are essential for maintaining homeostasis and regulating multiple cellular functions. mRNA, protein, and functional assays have confirmed the presence of all four P1 ADO receptors (A_1_, A_2A_, A_2B_, and A_3_), as well as P2 receptors including P2X1, P2X7, P2Y_2_, and P2Y_14_ [26, 27]. Meshki and colleagues reported expression of P2Y_2_, P2Y_4_, P2Y_6_, and P2Y_11_ receptors in human neutrophils by RT-PCR, but only P2Y_2_ receptor appeared functionally active [28]. Conversely, Scrivens and Dickenson demonstrated functional expression of P2Y_14_ receptor, which couples to inhibition of forskolin-induced cAMP accumulation and ERK1/2 activation, without affecting neutrophil degranulation [29]. Subsequent studies described functional roles for P2Y_6_ and P2Y_11_ receptors in neutrophils [30–32]. Collectively, these data highlight the need for further studies to clarify the expression patterns and functional relevance of nucleotide receptors in neutrophils.

Neutrophils are the first responders to arrive at the site of microbial invasion, migrating due to chemoattractant gradients that induce polarization and directed movement. Chen and colleagues showed that agonists of the formyl peptide receptor (FPR) on PMNs triggered the release of ATP, which facilitated downstream signaling events that were required for the activation of functional cell responses. They suggested that purinergic signaling processes represented potential targets for therapeutic approaches to modulate the responses of PMNs in inflammatory or infectious diseases [33]. Neutrophil activation induces a conformational change to “front and back” orientation. ATP binding to P2Y_2_ receptor guides forward movement, whereas hydrolysis of ATP to ADO by CD39 and CD73, followed by A_3_ and A_2A_ receptor engagement, provides inhibitory “back” signals that regulate positioning and migration speed [27]. Although both A_3_ and A_2A_ receptors modulate neutrophil chemotaxis, their roles are spatially and mechanistically compartmentalized within the polarized cell. At the leading edge (front), where actin polymerization drives membrane protrusion and directional sensing, ATP released in an autocrine manner is rapidly converted into ADO, which preferentially activates A_3_ receptors, promoting pro-migratory signaling pathways that enhance protrusion and forward movement. In contrast, at the trailing edge (uropod), where detachment from the substrate and cytoskeletal contraction are required, the accumulation of extracellular ADO engages A_2A_ receptors, triggering inhibitory and cAMP-dependent pathways that suppress excessive activation while facilitating rear retraction. Together, this spatially coordinated ATP–adenosine axis establishes a push–pull system, in which A_3_ receptor signaling drives frontness and A_2A_ receptor signaling supports backness, ensuring efficient and directed neutrophil migration. Neutrophil adhesion to the endothelium is promoted via A_1_ receptor activation and inhibited through A_2A_ receptor signaling [12]. Additional receptors involved in chemotaxis include A_3_, A_2A_, P2X1, P2Y_11_, and P2Y_14_ [2]. P2Y_6_ also contributes indirectly to chemotaxis, since it regulates the IL-8 release, a key chemokine recognized for neutrophils [27, 34].

Several steps of neutrophil recruitment—margination, rolling, adhesion, and crawling—appear to be influenced by purinergic signaling. In vivo studies indicate that P2 receptors, particularly non-neutrophil P2X1, contribute to recruitment [35]. Conversely, A_2A_ receptor stimulation reduces neutrophil rolling and adhesion in vitro [35, 36].

At inflammatory sites, neutrophils engage purinergic-dependent effector mechanisms regulating degranulation, chemotaxis, and antimicrobial activity. ATPγS, a non-hydrolyzable ATP analog, enhances fMLP-induced degranulation, whereas hydrolyzable ATP is converted to ADO by ectonucleotidases, inhibiting this response [37]. ADO also suppresses neutrophil superoxide (O₂⁻) production via A_2A_ and A_2B_ receptors and inhibits fMLP-induced degranulation. Phagocytosis and bactericidal activity are similarly inhibited. Interaction with A_2A_ receptors further suppresses cytokine and chemokine expression and release in response to LPS [38]. Activation of A_2A_ receptors can delay neutrophil aging and promote polarization to N2 phenotypes, with potential therapeutic implications in cancer, particularly in settings where A_2A_ receptor signaling contributes to immune suppression [39].

**Fig. 1:**
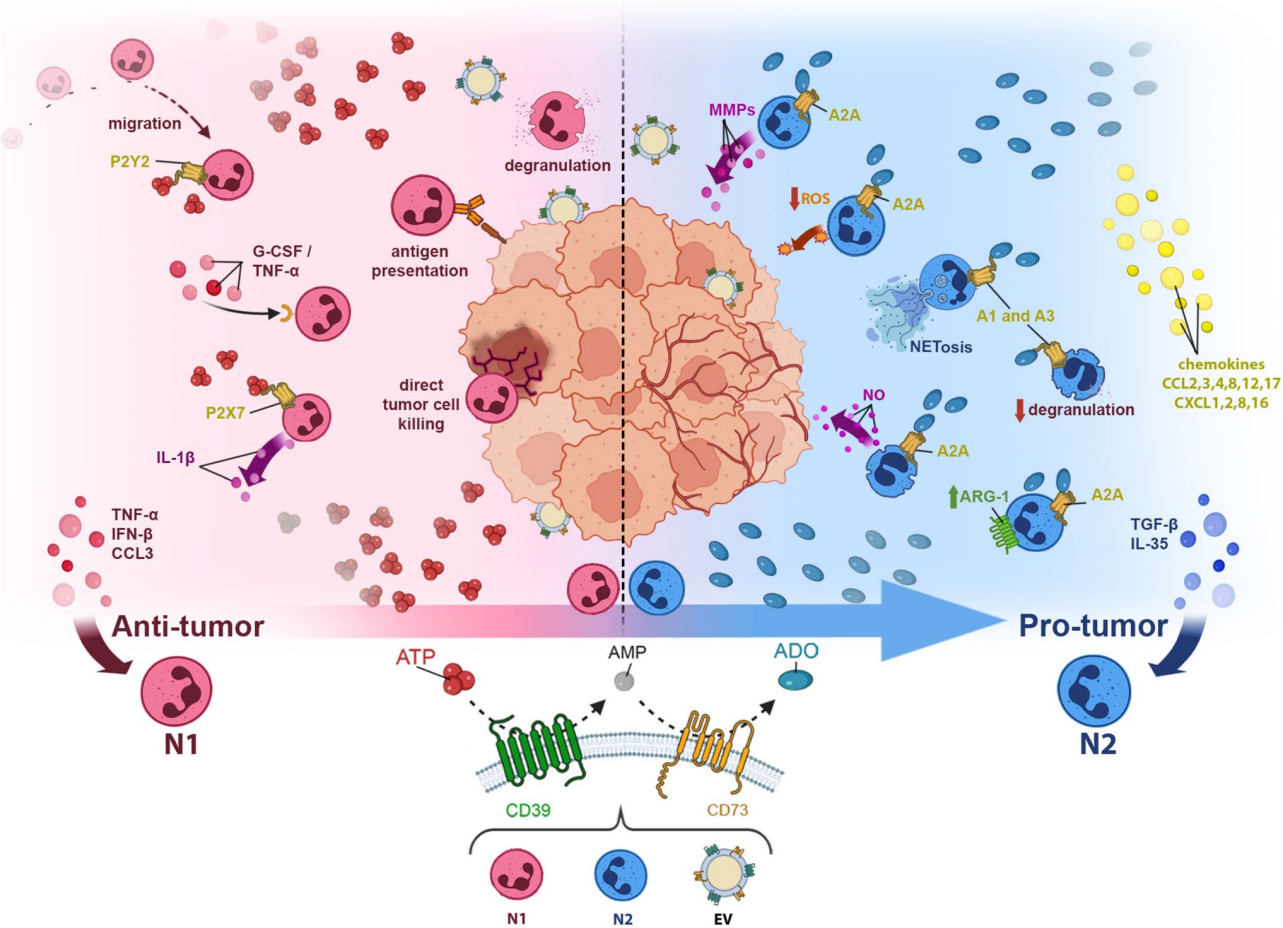
Purinergic and microenvironmental signals driving Tumor-Associated Neutrophil (TAN) polarization. Schematic overview highlighting the role of purinergic signaling in regulating TAN plasticity within the TME. TANs may initially acquire an antitumor N1 phenotype, characterized by the production of pro-inflammatory mediators such as TNF-α, CCL3, and increased ICAM-1 expression. This phenotype is supported by extracellular ATP, which acts as a danger-associated nucleotide signal that promotes neutrophil activation and pro-inflammatory functions. N1 TANs can exert direct tumor cell killing, recognize tumor-associated antigens, and eliminate tumor cells through degranulation and cytotoxic mediator release. ATP can act on P2X7 receptors to stimulate the production of pro-inflammatory cytokines such as IL-1β, while its action on P2Y_2_receptors is associated with the chemotaxis/migration of these cells to the tumor site. Within the TME, extracellular ATP is progressively hydrolyzed by the ectonucleotidases CD39 and CD73, leading to the accumulation of ADO. Increased ADO levels activate A_2A_ receptors on neutrophils, promoting a functional shift toward the immunosuppressive N2 phenotype. N2 TANs are associated with elevated chemokine production and higher ARG1 expression and contribute to tumor progression through the release of MMPs, ROS, and NO, which support tumor-associated angiogenesis. In addition, N2 neutrophils can form neutrophil NETs that facilitate metastasis and impair antitumor immune responses. Additional cytokine signals within the TME, including IFN-β, TGF-β, and IL-35, further modulate the balance between N1 and N2 polarization and their downstream effects on tumor progression

The effects of A_2A_ receptor signaling on neutrophil polarization must be interpreted within the context of ADO accumulation in TME. The upregulation and sequential activity of CD39 and CD73 promote an ADO-rich milieu. Under these conditions, sustained activation of A_2A_ receptors enhances favoring immunosuppressive and tumor-supportive phenotypes [39]. Thus, the N1 to N2 polarization shift is not merely a receptor-driven event, but rather a consequence of the metabolic reprogramming of the purinergic landscape, in which ADO production and availability critically shape neutrophil functional identity within the TME (Fig. 1).

P2X1 and P2X7 receptors also contribute to neutrophil antimicrobial activity during LPS-induced ATP signaling [42]. ATP released at inflammatory sites interacts with P2Y_2_ receptors on leukocytes, stimulating phagocytic clearance by neutrophils and macrophages and facilitating resolution of inflammation. P2X7 receptor activates NOD-like receptor inflammasomes, triggering pro-caspase-1 activation and IL-1ß and IL-18 release, mediating inflammatory and immune responses against pathogens or tumor cells [26, 43].

NET release, a critical antimicrobial mechanism, is modulated by purinergic signaling. P2Y_6_ is essential for NET formation in gout disease, whereas ADO promotes NET release via A_1_ and A_3_ receptors and inhibits it via A_2A_ [44–46]. The influence of purinergic signaling on neutrophil responses to infection and inflammation has implications in cancer, and neutrophil–tumor interactions are recognized.

Neutrophil-driven inflammation is a key contributor to tumor initiation, especially in chronically inflamed tissues. Purinergic signaling further amplifies this process, as extracellular nucleotides released from damaged cells activate P2Y_2_ receptor (by ATP) on neutrophils, enhancing their recruitment and activation. Activated neutrophils then release genotoxic mediators that induce epithelial DNA damage, fostering genomic instability and oncogenic transformation [47, 48].

Neutrophils, tumor cells, and stromal cells express the CD39/CD73 axis, whose coordinated activity converts extracellular ATP into adenosine, shifting the microenvironment from a pro-inflammatory to an immunosuppressive state [49]. This metabolic conversion attenuates antitumor immune responses, consistent with the well-established immunomodulatory and immunosuppressive effects ADO [50].

Furthermore, UDP-mediated P2Y_6_ receptor activation has been associated with increased IL-8/CXCL8 production in parenchymal and myeloid cells, supporting neutrophil recruitment and tumor infiltration, as well as enhanced NET formation [30, 51], linking purinergic signaling to neutrophil-driven tumor-promoting mechanisms [44]. These effects are discussed in greater detail in subsequent sections.

This dynamic purinergic balance not only regulates neutrophil-induced tissue injury and genomic instability but also shapes immune surveillance during the earliest phases of malignant transformation. The following sections will further detail how purinergic signaling pathways orchestrate neutrophil plasticity and contribute to tumor initiation. Understanding how purinergic receptor activation generates intracellular signaling in neutrophils may reveal novel therapeutic targets.

## An overview of purinergic signaling in cancer

Exacerbated cellular growth (increased proliferation), acquisition of an aggressive phenotype (metastatic dissemination), and immune modulation (immune evasion and suppression) are processes critically influenced by nucleotide and nucleoside metabolism, availability, and signaling within TME. Current evidence supports the concept that these mechanisms are tightly interconnected and collectively contribute to tumor progression, highlighting the purinergic system as a central regulatory axis in this context [52].

In neoplastic tissues, both de novo (purine and pyrimidine) and *salvage* nucleotide synthesis pathways are markedly upregulated to function as the primary source of nucleotide supply for newly formed cells. This metabolic adaptation ensures a continuous provision of substrates required for DNA and RNA replication, thereby sustaining rapid cell division [51]. Moreover, dysregulated nucleotide metabolism has been shown to accelerate tumor development while simultaneously impairing anti-tumor immune responses within the TME. Consistent with this metabolic rewiring, cancer cells preferentially enhance nucleotide biosynthetic pathways to meet the high anabolic demands of accelerated proliferation. Notably, supraphysiological levels of nucleotides (particularly GTP) directly fuel signaling cascades that drive tumor progression and metastatic dissemination [53].

Beyond intracellular metabolic adaptations, extracellular nucleotides and nucleosides exert a broad spectrum of biological effects. These actions depend not only on the enzymatic machinery that regulates their local concentrations but also on the expression profile and functional state of their respective purinergic receptors.

The TME drives increased extracellular ADO accumulation and up regulation of immunosuppressive A_2A_ and A_2B_ receptors on immune cells. Hypoxia-induced activation of A_2B_ supports maintenance of the tumor epithelial barrier, limiting infiltration of antitumor immune cells. Furthermore, A_2A_- and A_2B_-mediated immunosuppression facilitates metastatic progression in solid tumors. Elevated A_2B_ receptor expression and ADO signaling under hypoxic conditions have been implicated in tumor growth, angiogenesis, and immune evasion [54]. In ADO-rich TMEs, ADO-dependent activation of A_2A_ receptor triggers immunosuppressive pathways that hinder effective anticancer immunity [55].

Modulation of the A_2A_ receptor signaling axis has been shown to enhance T-cell function and antitumor responses. Pharmacological inhibition of A_2A_ receptor potentiates immune checkpoint therapy by strengthening CD8⁺ T-cell-mediated cytotoxicity [56]. ADO binding to A_2A_ or A_2B_ receptor activates adenylyl cyclase, increasing intracellular cyclic AMP (cAMP) levels, which suppresses antitumor immunity and promotes activation of immunosuppressive cell populations. Specifically, A_2A_ receptor signaling inhibits T-cell proliferation, activation, and cytokine secretion while promoting differentiation and expansion of Tregs. Accordingly, targeting A_2A_ receptor with negative allosteric modulators (NAMs) has emerged as a promising strategy to overcome limitations of competitive orthosteric antagonists [55].

Several solid tumors—including lung cancer, pheochromocytoma, hepatocellular carcinoma, bladder urothelial carcinoma, cervical squamous cell carcinoma, and gastric adenocarcinoma—show pronounced overexpression of ADO pathway components, particularly A_2A_ receptor and A_2B_ receptor, making them prime candidates for therapies targeting the extracellular adenosine (eADO) pathway. A_2A_ receptor antagonists, such as SCH58261 and PBF-509, restore T cell proliferation suppressed by mesenchymal stromal cells, thereby restoring antitumor immunity [32]. Similarly, pharmacological inhibition of A_2A_ receptor with ciforadenant, combined with PD-L1 blockade using atezolizumab, elicited clinical responses in patients with advanced metastatic castration-resistant prostate cancer refractory to checkpoint inhibitors [57].

Activation of the A_3_ receptor promotes antitumor immunity by inhibiting proliferation of multiple cancer cell types [58]. In neutrophils, eosinophils, and macrophages, A_3_ receptor signaling mediates anti-inflammatory effects by reducing degranulation and inducing anti-inflammatory cytokine production. A_3_ receptor agonists also exert cytostatic effects on tumor cells in vitro and in vivo, primarily through dysregulation of Wnt signaling [59]. Probenecid has been shown to selectively modulate P2Y_14_, A_2A_, and A_3_ receptors, influencing ERK/MAPK and PI3K/AKT pathways that control epithelial–mesenchymal transition, angiogenesis, invasion, and cytoskeletal remodeling in hepatocellular carcinoma, potentially impairing tumor-promoting signaling [60].

Extracellular vesicles (EVs) in plasma represent a mechanism for controlling excessive neutrophil activation via CD39 and CD73. These enzymes on EVs are metabolically active and modulate neutrophil activity through A_2A_ receptor signaling, highlighting their emerging relevance in cancer [61].

P2 purinergic receptors exhibit diverse and context-dependent roles throughout cancer development. Activation of specific P2 receptor subtypes can sustain inflammatory responses that, depending on the cellular and microenvironmental context, may contribute to tumor initiation and progression [62]. P2X1 receptor expression in neutrophils associated with gastric cancer enhances neutrophil survival, which inhibits gastric cancer cell migration, invasion, and viability, while promoting apoptosis [63]. Tumors may evade immunity by recruiting P2X1 receptor-negative neutrophils, which exhibit immunosuppressive phenotypes and drive PD-1-mediated CD8⁺ T-cell exhaustion. P2X1 receptor-deficient neutrophils infiltrate liver metastases of pancreatic ductal adenocarcinoma, promoting immunosuppressive microenvironments and metastatic colonization [64].

P2X4 receptor has been implicated in hepatocellular carcinoma progression by increasing calcium influx, activating PI3K/AKT signaling, and suppressing antitumor immunity [65]. Combined antagonism of P2X7 and A_2A_ receptors suppresses tumor growth in vivo, reduces metastasis, and decreases IL-17 and IL-23 production, emphasizing crosstalk among P2X7/CD39/CD73 and A_2A_ receptor in colon cancer metastasis [66]. P2X7 receptor signaling on immune cells regulates or responses in a context-dependent manner, with its functional outcome varying according to tumor type, disease stage, and the intensity and duration of extracellular ATP gradients within the tumor microenvironment. In parallel, endothelial P2Y_2_ receptor activation facilitates lymphocyte infiltration into inflamed tissues and, depending on the cellular compartment in which it is engaged, may promote metastatic progression by enhancing lysyl oxidase (LOX) activity and extracellular matrix cross-linking, thereby contributing to the establishment of a pre-metastatic niche [67].

P2Y_6_ receptor antagonism using MRS2578 inhibits cell proliferation and suppresses LPS-induced cytokine release in glioma cells. Elevated P2Y_14_ receptor expression has been observed in acute leukemia cell lines resistant to PKI-587 (a PI3K/mTOR inhibitor), correlating with poor overall survival in FLT3-ITD-positive AML and in acute lymphoblastic leukemia [68].

## Role of neutrophils and purinergic signaling in the tumor microenvironment

Tumor development often arises in the context of chronic inflammation [69]. Whether acting as an initiating factor or emerging during tumor progression, infiltrating immune cells are present in the TME as part of the host’s attempt to control cancer. Among these cells, neutrophils are key players. Evidence indicates that neutrophils can promote tumorigenesis by amplifying DNA damage through ROS release [70], enhancing cancer cell growth and proliferation via prostaglandin E2 [71] and elastase [72], or supporting metastasis through NET formation [73]. Neutrophils also contribute to tumor-associated secondary events, including cancer-related thrombosis and dysfunction of distant organs. Their activity is prominent during early tumor development, whereas the pronounced immunosuppressive features of PMN-MDSCs emerge predominantly in advanced stages of cancer [74].

Although neutrophils generally exhibit pro-tumor functions, they retain the ability to identify and eliminate cancer cells, reducing metastatic spread. Effective neutrophil cytotoxicity requires proper activation, targeted attraction, and specificity. Several soluble proteins released from neutrophil granules upon activation are associated with tumor progression and are considered potential biomarkers [75].

Neutrophil activation occurs via classical receptors, including Toll-like receptors (TLRs), NOD-like receptors (NLRs), and Fc gamma receptors (FcγRs). Tumor recognition may occur through direct contact or antibody-dependent mechanisms. For instance, tumors expressing TRPM2 are more susceptible to neutrophil-mediated cytotoxicity [76], and TNF-related apoptosis-inducing ligand (TRAIL) can induce cancer cell death [77].

Neutrophil responses are directly influenced by TME factors. TGF-β suppresses neutrophil antitumor activity and limits neutrophil infiltration in the tumor, whereas interferons (IFNs) recruit and activate neutrophils to exert antitumor effects. ATP and ADO are major TME components and can dictate the biased response of neutrophils in this environment. Extracellular ATP serves as a “find-me” signal, directing phagocytes to sites of infection or tumor cells [24, 26]. Tumor cells and tumor-associated stromal cells also release neutrophil-attracting CXC chemokines [78]. Neutrophil chemotaxis is further amplified by ATP release from neutrophils themselves, enhancing P2Y_2_ receptor signal to guide these cells [27]. Garcia-Rocha and colleagues demonstrated that ADO signaling induces TGF-β expression, which can be blocked by A_2A_ and A_2B_ receptor antagonism in cervical cancer [79]. In melanoma models, A_2A_ receptor blockade triggers compensatory feedback in ectonucleotidases, preserving extracellular ADO in the TME [80]

Extracellular ATP can activate the NLRP3 inflammasome via pannexin 1 (Panx1), which facilitates ATP release and P2X7 receptor signaling [43]. Panx1 plays context-dependent roles in tumor progression, acting as either tumor-promoting or tumor-suppressive depending on the cellular environment [81]. This pathway typically leads to IL-1β release and promotes antitumor immunity [26, 43]

In cancer, neutrophils adopt both antitumor (N1) and pro-tumor (N2) phenotypes, with tumor-associated neutrophils often exhibiting mixed characteristics [82]. In colorectal cancer, tumor-derived GM-CSF reprograms antitumor neutrophils into a pro-tumorigenic phenotype via the AKT–NF-κB–NFAT5 pathway. Upon stimulation with CXCR2 ligands, these neutrophils release NETs, which support tumor progression by enhancing cancer proliferation, suppressing antitumor immunity, and promoting liver metastasis [83].

An important mechanism involves neutrophil migration dependent on P2X1-type receptors. In multiple models of pancreatic ductal adenocarcinoma (PDAC) a subset of neutrophils lacking P2X1 receptor expression was identified infiltrating and accumulating within the TME of liver metastases, whereas neutrophils in adjacent liver tissues retained expression of this receptor [84]. Subsequent analyses demonstrated that P2X1⁻ neutrophils exhibited higher expression levels of the chemotactic receptor CXCR2 [60], compared with P2X1⁺ neutrophils, suggesting that enhanced CXCR2 expression contributes to the accumulation of P2X1⁻ neutrophils within the hepatic metastatic niche [84].

Transcriptomic analysis revealed distinct metabolic profiles between these neutrophil subpopulations. P2X1⁻ neutrophils showed increased expression of genes associated with mitochondrial metabolism, including the tricarboxylic acid cycle and fatty acid oxidation. In contrast, P2X1⁺ neutrophils displayed higher expression of genes related to glycolysis [84]. This metabolic divergence is consistent with the association of mitochondrial metabolism with an immunosuppressive phenotype, whereas glycolysis supports immunostimulatory functions [85].

Functionally, P2X1⁺ neutrophils expressed markers characteristic of N1-like neutrophils, increased ROS production, and PD-L1 expression, all of which are associated with antitumor activity. Conversely, P2X1⁻ neutrophils exhibited higher expression of N2-like markers linked to pro-tumorigenic activities [84]. Taken together, these findings indicate that P2X1 receptor signaling sustains the antitumor function of neutrophils, highlighting P2X1 receptor as a promising therapeutic target [86].

Extracellular nucleotides and nucleosides—ATP, UTP, and ADO—modulate neutrophil differentiation and function. While extracellular ATP can exert immunostimulatory effects, its functional impact in the TME depends on its concentration, temporal dynamics, receptor engagement, and the balance with ectonucleotidase activity. Chronic ATP release under conditions such as hypoxia promotes upregulation of CD39 and CD73, converting ATP into immunosuppressive and pro-tumoral ADO [40, 41, 87]. Consequently, purinergic signaling in the TME exerts complex and context-dependent effects: ADO acting through P1 receptors is generally associated with immunosuppressive and tumor-promoting mechanisms, whereas ATP signaling through specific P2 receptor subtypes may exert either pro- or antitumoral effects depending on receptor subtype, ATP concentration, and cellular context*.* Nevertheless, the specific roles of purinergic signaling in neutrophils during cancer remain incompletely understood.

In PMN-MDSCs, ATP signaling can paradoxically be immunosuppressive. P2X7 receptor activation in these cells increases ROS, arginase-1, and TGF-β release, promoting genetic instability, inhibiting T-cell proliferation and interferon production, and favoring N2 polarization [10, 88–92].

Evidence supports a pro-tumorigenic UDP/P2Y_6_/IL-8/NET axis, as UDP-driven P2Y_6_ activation enhances IL-8 release from monocytes, promoting neutrophil recruitment and increased NET density within the tumor microenvironment [30, 51]. Moreover, tumor-cell P2Y_6_ expression is associated with elevated IL-8 levels, neutrophil infiltration, and tumor proliferation, including in glioma [93]. In addition, Sil and colleagues reported that pharmacological antagonism of P2Y_6_ receptor (e.g., MRS2578) can limit neutrophil activation and NET formation [44]. In a murine lymphoma model, P2Y_6_ receptor inhibition altered cell-cycle regulatory proteins, induced cell-cycle arrest, and suppressed tumor growth; blockade of the UDP/P2Y_6_ axis also decreased neutrophil recruitment to the TME and reduced metastatic spread [94, 95]. Important caveats remain: MRS2578 may have off-target or cytotoxic effects, P2Y_6_ receptor expression and function in neutrophils are incompletely characterized, and tumors exert systemic influences on NET production while NETs reciprocally remodel the TME and facilitate metastasis [5, 96–98]. However, P2Y_6_ receptor signaling has been associated with inhibition of neutrophil apoptosis, which could prolong neutrophil persistence in tumors [98]. Together, these data point to a highly context-dependent role for P2Y_6_ receptor in cancer and underscore the need for cell-specific, quantitative studies of receptor expression, downstream signaling, and local UDP/purinergic gradients to define when P2Y_6_ receptor blockade will be therapeutically beneficial. The main findings of key studies investigating purinergic receptors signaling in neutrophil function and tumor progression are summarized in Table 1.

**Table 1:** Overview of key studies investigating purinergic receptor signaling in neutrophil function and tumor progression

Reference	Study design	Findings
Nagaoka, 2010	In vitro (human neutrophils)	**Purinergic target:** P2Y_6_ receptor. **Tumor model:** Not directly assessed. **Direct tumor effect:** Not evaluated. **Neutrophil effect:** P2Y_6_ receptor activation inhibited neutrophil apoptosis, prolonging cell survival. **Overall interpretation:** Sustained neutrophil persistence may indirectly contribute to chronic inflammatory conditions within the TME
Wawrzyniak, 2013	In vitro/metabolic mechanistic analyses	**Purinergic target:** nucleotide metabolic pathways (elevated GTP). **Tumor model:** cancer cell metabolic studies. **Direct tumor effect:** supraphysiological nucleotide levels promoted signaling pathways supporting tumor progression and metastasis. **Neutrophil effect:** not directly studied. **Overall interpretation:** metabolic reprogramming of nucleotide pools supports tumor growth and may reshape the TME
Qin, 2020	In vivo (murine B16F10 melanoma lung metastasis model)	**Purinergic target:** P2Y_6_ receptor (UDP). **Tumor model:** experimental lung metastasis. **Direct tumor effect:** P2Y_6_ receptor deficiency significantly reduced pulmonary metastatic burden. **Neutrophil effect:** P2Y_6_ receptor promoted neutrophil recruitment to the pre-metastatic niche. **Overall interpretation:** P2Y_6_-driven neutrophil infiltration facilitates metastatic colonization
Wang, 2021	In vivo (murine PDAC liver metastasis model)	**Purinergic target:** P2X1 receptor. **Tumor model:** hepatic metastasis. **Direct tumor effect:** P2RX1 deficiency enhanced metastatic progression. **Neutrophil effect:** loss of P2X1 receptor shifted neutrophils toward an N2-like immunosuppressive phenotype (↑Arg1, ↑MMP-9, ↑PD-L1; ↓ROS). **Overall interpretation:** P2RX1 signaling maintains antitumoral neutrophil activity and restricts metastasis
Zhang, 2021	In vivo + human samples	**Purinergic target:** P2X7 receptor. **Tumor model:** solid tumor inflammatory microenvironment. **Direct tumor effect:** P2X7 receptor activation associated with enhanced tumor progression. **Neutrophil effect:** increased production of pro-inflammatory cytokines (e.g., IL-1β), contributing to tumor-supportive inflammation. **Overall interpretation:** P2X7-mediated inflammatory signaling sustains a pro-tumorigenic TME
Roskov, 2022	In vivo + in vitro (tumor microenvironment studies)	**Purinergic target:** P2Y_12_ receptor (platelet-neutrophil axis). **Tumor model:** metastatic tumor models. **Direct tumor effect:** enhanced tumor progression and metastatic niche support. **Neutrophil effect:** purinergic signaling facilitated neutrophil activation within platelet-mediated interactions. **Overall interpretation:** P2Y_12_-dependent cross-talk supports metastasis through immune modulation
Mullen, 2023	In vitro + molecular analyses	**Purinergic target:** nucleotide biosynthesis pathways. **Tumor model:** cancer cell metabolic studies. **Direct tumor effect:** upregulated nucleotide synthesis sustained rapid proliferation and genomic replication. **Neutrophil effect:** not directly evaluated. **Overall interpretation:** nucleotide metabolic rewiring supports tumor growth and indirectly alters TME dynamics
Pegoraro, 2025	In vivo + human data	**Purinergic target:** P2X7 receptor. **Tumor model:** solid tumor models. **Direct tumor effect:** P2X7 receptor signaling promoted tumor progression via sustained inflammation. **Neutrophil effect:** elevated neutrophil-derived cytokines (notably IL-1β) enhanced tumor-permissive inflammation. **Overall interpretation:** P2X7 receptor amplifies inflammatory circuits that favor tumor development

Moreover, blockage of the UDP/P2Y_6_ receptor prevented tumor metastasis and reduced neutrophils recruitment to TME in lung cancer [95]. Nevertheless, the exclusive reliance on MRS2578 warrants further validation through complementary approaches, such as genetic knockdown or alternative pharmacological tools, to confirm the involvement of P2Y_6_ receptor. Thereby, P2Y_6_ receptor may represent a promising therapeutic target to avoid the NET formation and metastasis, but these conclusions require validation to ensure their translational relevance.

**Fig. 2:**
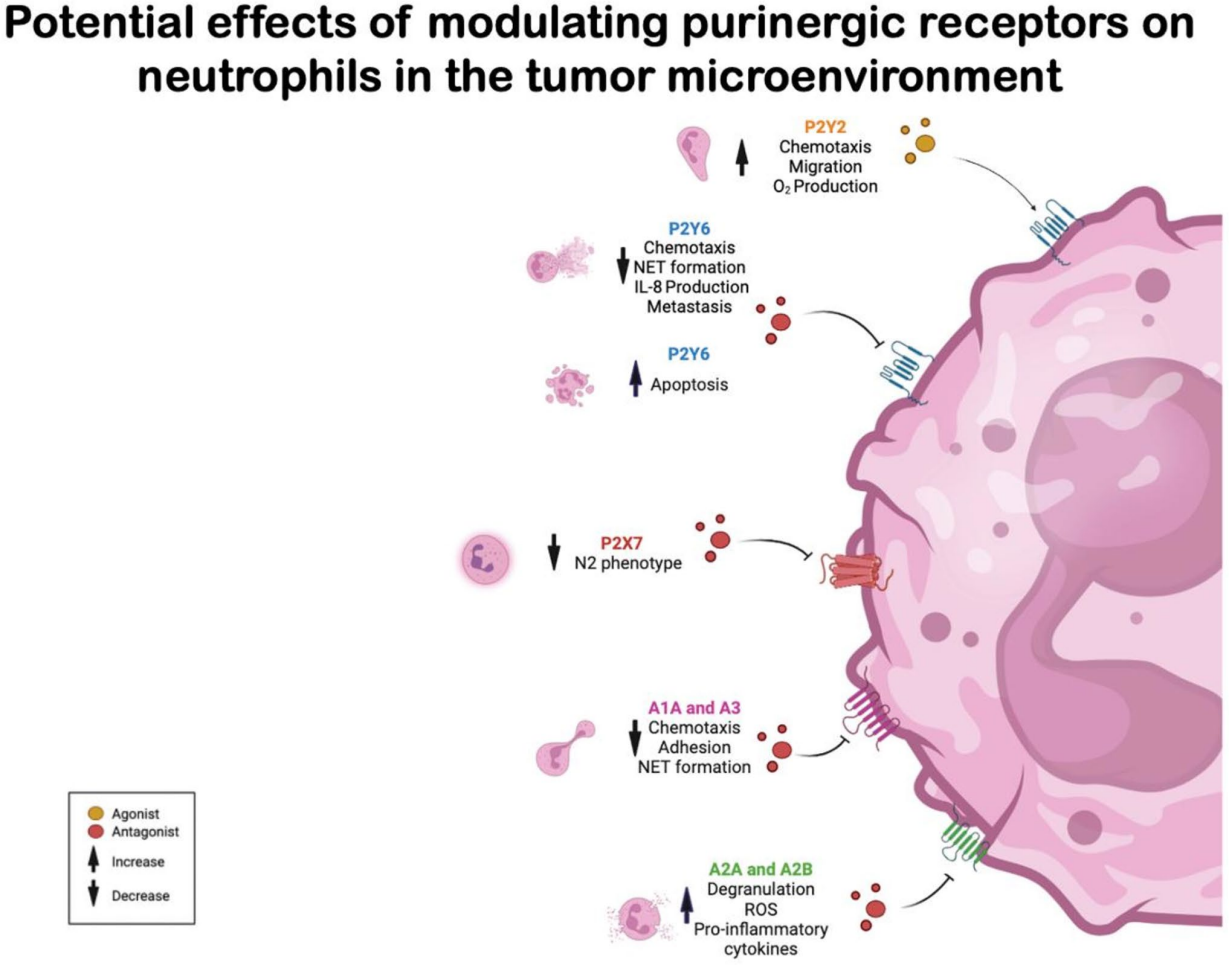
Potential effects of modulating purinergic receptors on neutrophils in the tumor microenvironment. The impact of antagonists or agonists depends on the specific functions they target. However, the outcomes may vary based on the conditions under which they are applied. In the tumor microenvironment (TME), we need a robust defense against tumor cells, prevention of senescence, improvement of the phenotypic profile of pro-tumoral populations, and even inhibition of metastasis

Sustained elevations in ADO exert a significant regulatory influence on neutrophil function. Engagement of A_1_ and A_3_ receptors promotes adhesion, chemotaxis, and NET formation. In contrast, activation of the A_2A_ receptor mediates anti-inflammatory effects by suppressing ROS production, degranulation, and pro-inflammatory cytokine release. Notably, despite its predominantly inhibitory profile, A_2A_ receptor signaling also contributes to the spatiotemporal coordination of neutrophil migration by supporting cell polarization and facilitating uropod retraction, thereby integrating into the push–pull mechanism that underlies efficient directed motility [99–102]. Additionally, ADO signaling through A_1_ and A_3_ receptors stimulates the release of NETs while inhibiting A_2A_ receptor signaling [46]. The balance of P1 receptor signaling in neutrophils depends on receptor expression levels and affinity. Uniform A_2A_ receptor expression generates inhibitory signals that modulate chemotaxis and avoid excessive activation. ADO signaling through A_2A_ receptor also induces TGF-β, promoting neutrophil immunosuppressive activity, and modulating the TME. Extracellular ADO levels are further regulated by CD39, CD73, and adenosine deaminase (ADA) activities, and this regulation consists in an important factor to TME modulation [49]. Several possible therapeutic targets, involving purinergic signaling on neutrophils, are represented in Fig. 2.

## Therapeutic strategies

Damage-associated molecular pattern molecules are released by injured tissue and by cancer cells to initiate adaptive and innate immune responses. These molecules include purines such as ATP, which mediates inflammasome activation and activation of dendritic cells, potentiating antitumor response. Strategies for manipulating the tumor microenvironment with purinergic receptor antagonists or inhibition of ectonucleotidases have demonstrated therapeutical potential in cancer treatment [103].

The multifaceted roles of neutrophils make modulating their functions a complex task. Therapeutic strategies require fine-tuning to enhance antitumor activity while limiting deleterious effects during inflammation. NETosis has emerged as a potential therapeutic target for limiting metastatic dissemination. The entrapment of tumor cells within NETs impairs the cytotoxic activity of CD8⁺ T cells by preventing direct contact between tumor cells and cytotoxic T lymphocytes (CTLs). Currently, several clinical trials are underway to evaluate the efficacy of CXCR1/CXCR2 inhibitors in blocking NET formation and controlling metastatic progression [51].

ADO signaling through P1 receptors regulates neutrophil-mediated inflammation. A_1_ and A_3_ receptors enhance chemotaxis, whereas A_2A_ and A_2B_ receptors engagement suppresses phagocytosis, degranulation, leukocyte recruitment, and neutrophil adhesion to endothelium [49, 99, 100]. Neutrophils express all four P1 receptors as well as CD39 and CD73, enabling rapid conversion of released ATP into ADO, particularly in the TME, where they modulate the inflammatory response. Under homeostatic conditions, neutrophils secrete low levels of ADO; however, upon activation, they can become a major source of ADO at inflammatory sites [45]. Moreover, a wide range of cancers show a worse prognosis when associated with a high number of neutrophils in the microenvironment [104]. The immune constitution of TME is exceptional, and in conjunction with soluble factors, such as ADO, may indicate prognosis or therapeutic potential. Considering receptor affinity, ADO may initially exert immunostimulatory effects on neutrophils, followed by sustained inhibitory signaling.

Therapeutic strategies targeting the CD39/CD73 axis (blockade or ablation) have been studied, which aim to stimulate inflammatory and antitumor pathways, reducing ADO-mediated immunosuppression [97]. Although there are some encouraging results, few studies demonstrate the effects of this blockade on the effector function of neutrophils. But, despite the planned effects, the blockade, stimulation or inhibition of some enzyme or receptor could generate effects not initially considered. On the other hand, neutrophil plasticity (N1 vs. N2) allows these cells to adopt pro- or antitumoral roles in response to soluble factors such as ATP, UDP, ADO, TGF-β, and IFNs, which may serve as therapeutic targets.

Although the phenotype of tumor-infiltrating neutrophils is shaped by the microenvironment—including tumor type, stage, genetic background, and soluble mediators—differences in ectonucleotidases or purinergic receptor expression in these cells have not been consistently reported. Therefore, therapeutic approaches may focus on altering the environment, for instance by reducing ADO production and TGF-β levels through CD39/CD73 inhibition, or by blocking A_2A_ receptor signaling, which exerts broad immunosuppressive effects [105].

In parallel, P2X7 receptor agonism may be strategically explored to enhance innate immune activation, provided that ATP availability and cellular metabolic fitness support a pro-inflammatory and antitumoral response.

The dual role of P2X7 receptor signaling in cancer reflects the context-dependent nature of extracellular ATP sensing. In this framework, P2X7 receptor can be regarded as a bioenergetic- and cell state-dependent inflammatory modulator, integrating extracellular ATP gradients with the metabolic and differentiation status of immune and tumor cells. While sustained low-level activation within the tumor microenvironment may favor immunosuppressive myeloid polarization (e.g., ARG1 expression, TGF-β production, N2-like features), robust or acute P2X7 receptor activation can trigger inflammasome assembly, promote tumor cell death, and enhance antitumor immunity [43, 102, 106].

As discussed above, the outcomes resulting from ATP signaling through ionotropic or metabotropic receptors, as well as those arising from the upregulation and sequential activity of CD39/CD73—leading to increased production and signaling of ADO via P1 receptors—do not depend solely on the isolated presence of a specific receptor. Rather, they are primarily determined by the ATP:ADO ratio, its spatiotemporal dynamics within the microenvironment, and the relative expression of CD39, CD73, and ADA in tumor cells, immune cells, and EVs. Collectively, these factors modulate neutrophil plasticity and ultimately dictate whether these cells adopt pro-inflammatory/antitumoral phenotypes or immunosuppressive and tumor-promoting profiles.

Preclinical models and early-phase clinical trials have evaluated chemokine receptor targeting in combination with anti-PD-1 therapy (pembrolizumab), demonstrating effects on several immune populations. CXCR4 antagonism (AMD3100) enhanced anti-PD-1 efficacy by reducing neutrophil infiltration. In a phase II trial (NCT02826486), combining anti-PD-1 with CXCR4 inhibitor BL-8040/BKT140 decreased neutrophil infiltration and increased cytotoxic T-cell activity. CXCR2 inhibition prevented neutrophil recruitment and NET formation, while CXCR4 blockade promoted neutrophil IL-18 production, enhancing NK cell-mediated antitumor responses [107].

Purinergic-targeted strategies may complement these approaches. For example, P2X1 receptor antagonists could reduce neutrophil infiltration in the TME. Moreover, P2Y_12_ receptor inhibition by clopidogrel has been evaluated with anti-PD-1 and acetylsalicylic acid (NCT03245489), in relation to their antiplatelet effects, highlighting the therapeutic potential of targeting purinergic pathways in cancer [108]. Considering purinergic signaling in neutrophil activation, P2Y_6_ receptor blockade represents a promising strategy to prevent NET formation, limit metastasis, and enhance immunotherapy. Importantly, interventions must preserve neutrophil viability and prevent exhaustion or premature apoptosis, maintaining their physiological functions.

## Concluding remarks

Current evidence on purinergic signaling in tumor-associated neutrophils highlights the functional dichotomy of these cells in cancer. Neutrophil polarization and the effects of pathway activation are context-dependent, influenced by tumor type, stage, and the local purinergic landscape. Predicting neutrophil behavior in the TME remains challenging, and inappropriate modulation may lead to undesired effects. Soluble factors present before, during, and after tumor development—including those derived from the purinergic system—play a critical role in neutrophil polarization, determining their functional responses to cancer.

This discussion underscores the significant gaps in our understanding of purinergic signaling in neutrophils within tumors. Further studies are needed to elucidate how nucleotide signaling influences neutrophil function and tumor progression, and to determine whether targeting these pathways can be harnessed safely and effectively for therapeutic benefit.

## Data Availability

No datasets were generated or analysed during the current study.
